# Oxidative Stress Mechanisms Underlying Parkinson’s Disease-Associated Neurodegeneration in *C. elegans*

**DOI:** 10.3390/ijms141123103

**Published:** 2013-11-21

**Authors:** Sudipta Chakraborty, Julia Bornhorst, Thuy T. Nguyen, Michael Aschner

**Affiliations:** 1 Neuroscience Graduate Program, Vanderbilt University Medical Center, Nashville, TN 37232, USA; E-Mail: sudipta.chakraborty@vanderbilt.edu; 2 Center in Molecular Toxicology, Vanderbilt University Medical Center, Nashville, TN 37232, USA; E-Mail: thuy.t.nguyen@vanderbilt.edu; 3 Department of Pediatrics, Vanderbilt University Medical Center, Nashville, TN 37232, USA; E-Mail: bornhorst@uni-muenster.de; 4 Department of Pharmacology, Vanderbilt University Medical Center, Nashville, TN 37232, USA

**Keywords:** oxidative stress, neurodegeneration, Parkinson’s disease, *C. elegans*, DJ-1, Parkin, PINK1, Nrf2

## Abstract

Oxidative stress is thought to play a significant role in the development and progression of neurodegenerative diseases. Although it is currently considered a hallmark of such processes, the interweaving of a multitude of signaling cascades hinders complete understanding of the direct role of oxidative stress in neurodegeneration. In addition to its extensive use as an aging model, some researchers have turned to the invertebrate model *Caenorhabditis elegans* (*C. elegans*) in order to further investigate molecular mediators that either exacerbate or protect against reactive oxygen species (ROS)-mediated neurodegeneration. Due to their fully characterized genome and short life cycle, rapid generation of *C. elegans* genetic models can be useful to study upstream markers of oxidative stress within interconnected signaling pathways. This report will focus on the roles of *C. elegans* homologs for the oxidative stress-associated transcription factor Nrf2, as well as the autosomal recessive, early-onset Parkinson’s disease (PD)-associated proteins Parkin, DJ-1, and PINK1, in neurodegenerative processes.

## 1. Introduction

The prevalence of neurodegenerative disorders, such as Parkinson’s disease (PD), has been increasing at a disconcerting rate. As age is the most significant risk factor for the development of this and other neurological diseases, longer lifespan worldwide has resulted in an increased global burden that is both financial and emotional in nature. In addition to expensive costs for treatments that do not fully resolve all symptoms, the quality of life for ailing patients is poor. Moreover, caretakers are faced with the challenge of providing support to patients suffering from the disease, which inevitably takes a significant toll on their own financial, physical and emotional well being. Significant headway has been made towards elucidating the etiology of PD, characterized by the hallmark loss of dopaminergic (DAergic) neurons in the substantia nigra pars compacta (SNpc) region of the brain [1]. This selective cell loss results in both motor and cognitive deficits, with cardinal symptoms of bradykinesia, rigidity, tremor and postural instability that are accompanied by emotional and cognitive problems [2]. However, the true mechanisms behind PD pathophysiology remain unknown, resulting in unsustainable treatment options that only provide symptomatic relief and do not target the original cause of the disease.

While the majority of PD cases are sporadic in nature (idiopathic PD or IPD), about 10%–20% of cases are well documented as having genetic causes. Many PD-associated genes have been identified, including *DJ-1*, *PINK1*, *PARKIN*, *NURR1*, *LRRK2*, *UCH-L1*, and *SNCA* [2]. Despite the known functions of these PD-associated genes, their role within interconnected signaling pathways involved in PD pathogenesis remains highly complicated and not yet fully understood. Interestingly, a shared role among many of these disease genes is participation in oxidative stress pathways. In fact, oxidative stress is thought to be one of the primary mechanisms behind the onset and progression of the DAergic-specific neurodegeneration in PD [3], as highly neurotoxic free radicals are generated both through the metabolism of dopamine and its own auto-oxidation that can be enhanced by exposure to environmental stressors [4]. Both increased oxidative stress and mitochondrial dysfunction have been shown in various PD studies. In particular, patients have disrupted iron (Fe) metabolism, as well as altered mitochondrial energetics, with a decrease in mitochondrial complex I levels and overall oxidative phosphorylation in the substantia nigra (SN) [5]. Moreover, depletion of the antioxidant glutathione (GSH) is also a prominent molecular consequence in PD, with several recent studies focusing on the potential therapeutic benefits of GSH administration [6]. Together, these effects suggest an active and significant role of reactive oxygen/nitrogen species (ROS or RNS) generation in the DAergic neurodegeneration of PD. However, the exact role of oxidative stress and its timing within the pathogenesis and progression of the neurodegeneration remains an enigma, with the ever-growing list of PD-associated genes and proteins further cluttering the picture.

Many investigations into the pathophysiology behind the neurodegeneration seen in PD have focused on using rodents as a vertebrate model system. However, while their genome shows similarity to the human genome, the intricacies of the vertebrate brain have hampered fast progression in understanding the disease. For this reason, many researchers have turned to the genetically amenable *Caenorhabditis elegans* (*C. elegans*) model system. This invertebrate model contains the DAergic machinery necessary to study PD-associated neurodegeneration, as well as possessing transparent bodies that allow for *in vivo* visualization of neurons. In this review, *C. elegans* studies involving oxidative stress mechanisms associated with DAergic neurodegeneration will be discussed, including a special focus on the autosomal-recessive, early-onset PD-associated genes *DJ-1*, *PINK1*, *PARKIN*, and the major oxidative stress modulator Nrf2.

## 2 *. C. elegans* as a Model for Neurodegeneration

**Figure 1:**
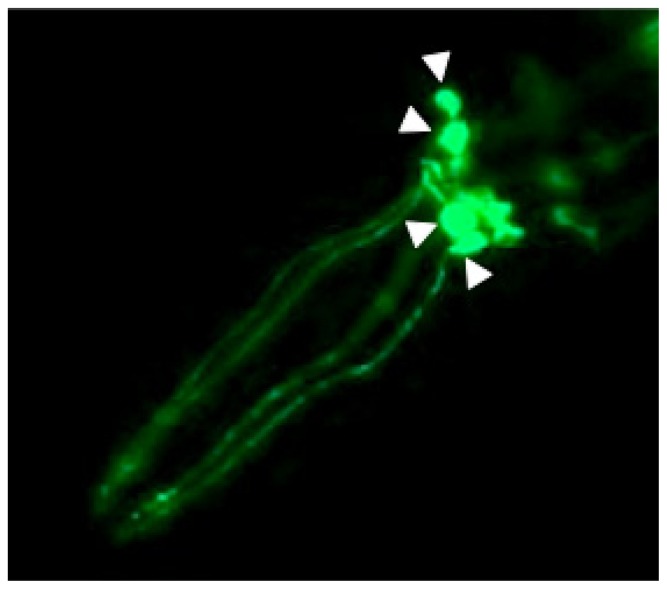
Arrowheads indicate the four dopaminergic cephalic (CEP) neurons in the head of the worm, with dendritic processes extending down to the tip of the nose.

The invertebrate *C. elegans* model provides several appealing advantages to investigate the connection between oxidative stress and DAergic neurodegeneration in PD. While these nematodes do not possess a brain, they do contain all necessary genetic information encoding components of the DAergic pathway [7]. This includes the homologs of the dopamine reuptake transporter (DAT, or DAT-1 in worms) [8]; the vesicular monoamine transporter 2 (VMAT2, or CAT-1 in worms) [9]; tyrosine hydroxylase (TH, or CAT-2 in worms) [10]; and dopamine receptors (D1 and D2-like receptors, or DOP-1 through DOP-4 in worms) [11]. Out of the total 302 neurons, hermaphroditic worms possess eight DAergic neurons: four CEP (cephalic) (Figure 1) and two ADE (anterior deirid) neurons in the head, as well as two PDE (posterior deirid) neurons in the tail [7]. Male worms contain six additional DAergic neurons in the tail. Neurons can be visualized through their transparent bodies using a fluorescent reporter, like GFP, that can be driven under the dat-1 promoter (e.g., p_dat-1_::GFP) [12]. Through the use of fluorescent and confocal microscopy, degeneration can be visualized by the presence of puncta and blebbing along dendritic processes; shrinking of soma; dendritic strand breaks; and loss of soma and dendrites.

Moreover, the *C. elegans* genome has been fully characterized [13], allowing for ease in studying genetic models of PD and DAergic neurodegeneration. Especially with a short lifespan (two to three weeks) and a quick life cycle (three days), the ease in unbiased, forward genetic screens has made *C. elegans* an attractive model to study neurodegeneration in PD [14]. Nematodes are first mutagenized to induce DNA mutations, followed by the isolation of animals with distinctive phenotypes of interest. In terms of PD, these phenotypes typically involve altered DA neuronal morphology or a DA-specific behavior, like the basal slowing response (alterations in body-bending behavior in response to food availability) [10]. Genetic mapping of progenies showing the modified trait is used to determine the location of the altered loci included [3]. As *C. elegans* reproduce quickly to generate 200–300 worms in one brood, genetic screens involving large numbers of animals can be performed within a relatively short amount of time [5].

Alternatively, reverse genetics is also a simple approach to study the effects of a specific gene of interest that may be involved in neurodegeneration. Transgenesis in worms is typically accomplished through microinjection and bombardment techniques. The former involves microinjecting a plasmid containing the regulatory sequence of the gene of interest fused to a fluorescent reporter that can later be used as a readout for that gene. Similarly, subcellular targeting sequences (such as the nuclear localization signal, NLS; or the mitochondrial targeting sequence, MTS) can drive protein localization, while cell or tissue-specific promoters (e.g., the aforementioned p_dat-1_::GFP transgene) can also be included in the plasmid to drive targeted gene expression. However, microinjection usually results in an unstable, extrachromosomal array, forcing the subsequent use of ultraviolet (UV) or gamma irradiation to integrate the transgene [15]. A second technique involves microparticle bombardment, otherwise known as biolistic transformation. Although this results in low-copy expression, the desired transgene is integrated into the genome [16].

In order to produce gene knockdowns, RNA interference (RNAi) is commonly employed in *C. elegans* [17]. RNAi in worms involves a systemic gene-knockdown that is dependent on RNA-dependent RNA polymerases (RdRPs) and can propagate in the F1 progeny of RNAi-exposed animals [18]. While originally conducted by microinjecting plasmids containing double-stranded RNA (dsRNA) specific to the target gene, other delivery methods include feeding worms bacteria expressing the dsRNA of interest [19] or soaking them in a dsRNA-containing solution [20]. While this technique is limited in expression specificity (some cells, such as neurons, are resistant to RNAi), there are RNAi-sensitive strains that allow for more effective knockdown (e.g., *rrf-3* and *eri-1* backgrounds) [21]. However, one must note that RNAi does not equally affect all tissues, along with the inherent variation in knockdown between dsRNA-fed animals. In addition to RNAi-mediated knockdown, knockout animals were formerly produced using randomized chemical mutagenesis, followed by screening for loss-of-function mutants using primers specific for deleted regions in target genes. However, a more targeted method was created using the Mos1 transposase, which cuts DNA at the location of the specific gene of interest [22]. The availability of knockout animals through the *Caenorhabditis* Genetics Center (CGC) is possible due to distribution of isolated deletion mutants from both the National BioResource Project of Japan (NBRP) and the *C. elegans* Gene Knockout Consortium (GKC) [23].

## 3. Assessing Oxidative Stress in *C. elegans*

### 3.1. Neurotoxins: PD-Mimetics

Some well-known pharmacological PD models in mammalian systems include the classical and highly selective neurotoxin 6-hydroxydopamine (6-OHDA), as well as 1-methyl-4-phenyl-1,2,3,6-tetrahydropyridine (MPTP) and its metabolite, MPP^+^ (1-methyl-4-phenylpyridinium ion). These toxins result in decreased ATP production, increased ROS production, and increased apoptosis of DAergic cells [24]. Similarly, herbicides and pesticides such as rotenone, paraquat, and maneb are also commonly used as PD models that result in increased ROS production and altered mitochondrial energetic [25]. A newer pharmacological model involves utilization of lipopolysaccharide (LPS), an inflammagen that causes production of RNS in DAergic cells via microglial activation, as another molecular feature of PD is nigrostriatal inflammation [26]. Similarly, in *C. elegans*, exposure to pesticides, 6-OHDA and MPTP/MPP^+^ all result in increased oxidative stress, ATP depletion, and disrupted mitochondria that appear with the hallmark DAergic neurodegeneration [27–29]. Although the use of LPS as a PD model has not been well studied in worms, the role of other environmental toxins, such as various heavy metals, has recently emerged in *C. elegans* literature. As an example, manganese (Mn) exposure results in DAergic neurodegeneration that is associated with increased oxidative stress [30]; with exposure to antioxidant compounds reversing the Mn-induced ROS induction [31]. Therefore, several studies have now validated the use of the *C. elegans* model system to study the role of oxidative stress in DAergic neurodegeneration.

In studying PD, 6-OHDA is of particular interest due to its endogenous nature as a neurotoxic metabolite of DA. The endogenous production of 6-OHDA is suggested to occur via a non-enzymatic reaction between DA, hydrogen peroxide and free iron at physiological concentrations. It is also hypothesized that *in vivo* production of 6-OHDA is more likely to occur in the highly oxidizing environment of DA neurons [32,33]. 6-OHDA generates an increase in the production of hydrogen peroxide and free radicals, including the superoxide ion and hydroxyl radical [34,35]. These reactive oxygen species are most likely generated through the non-enzymatic breakdown of 6-OHDA or direct inhibition of complex I and IV of the mitochondrial electron transport chain [34,36,37]. The resulting ROS production from 6-OHDA breakdown leads to lipid peroxidation, protein denaturation, and increases in glutathione, which are analogous to hallmarks found in postmortem PD patients [38]. The specificity of 6-OHDA for DA neurons is due to its affinity for the Na^+^- and Cl^−^-dependent DAT [39]. Therefore, characterization of the *C. elegans* model of DA neurodegeneration provides an opportunity to explore fundamental questions concerning the regulation of DAT. The 6-OHDA sensitivity of the DA neurons also provides an opportunity to examine the role various endogenous and exogenous compounds, as well as proteins involved in the biosynthetic pathways of DA neurotransmission, may play in normal DA neuron function and disease.

### 3.2. Measuring ROS Production *in Vivo*

Considering the amount of evidence that gives rise to the theory that oxidative stress is a key factor in neurodegeneration, studying oxidative damage will bring insights to our knowledge of disease. There are several ways to assess the role of oxidative damage in neurodegeneration; one could measure levels of ROS in whole worms and in isolated mitochondria, or measure indices of oxidative damage such as measuring levels of lipid and DNA oxidation products. To investigate the role of ROS in neurodegeneration, the first logical step would involve measuring levels of intraworm ROS. Several analytical approaches (EPR, chemiluminescence, fluorescence) have been used to detect ROS. Fluorescent or chemiluminescent dyes, such as dichlorodihydrofluorescein (DCFH-DA), dihydroethidium, and dihydrorhodamine are used frequently for measuring hydrogen peroxide, superoxide, and peroxynitrite either in whole worm or isolated mitochondria [40–42]. DCFH-DA is the most widely used probe for detecting intracellular H_2_O_2_ and oxidative stress due to its user-friendly nature. It is cell-permeable and is hydrolyzed intracellularly to the DCFH carboxylate anion, which is retained in the cell. Two-electron oxidation of DCFH results in the formation of a fluorescent product, dichlorofluorescein (DCF), which can be monitored by several fluorescence-based techniques, such as fluorescent microplate readers, confocal microscopy, and flow cytometry. However, the intracellular redox chemistry of DCFH is complex, and there are several limitations and artifacts associated with the DCF assay [43–45]. DCFH does not directly react with H_2_O_2_ to form the fluorescent DCF product; therefore, DCF fluorescence cannot be used as a direct measure of H_2_O_2_. Several oxidizing species will oxidize DCFH to DCF, making the dye nonspecific for a particular form of ROS. Additionally, the intermediate radical, DCF^•−^, rapidly reacts with O_2_ to form superoxide (O_2_^•−^). The dismutation of O_2_^•−^ yields additional H_2_O_2_[46], which can establish a redox-cycling mechanism leading to artificial amplification of the fluorescence signal intensity. In addition, one cannot assume that control and experimental samples exhibit the same efficiency in DCF radical generation; thus, linearity should not be assumed between different treatment conditions. Therefore, it is important to recognize the limitations of the dyes and avoid erroneous interpretations; DCFH-DA probe cannot reliably measure intracellular H_2_O_2_ and other reactive oxygen species, but instead may be more appropriate in use as a redox indicator probe that responds to oxidative insult [47].

Recent advances in constructing genetically encoded redox-sensitive sensors have opened up new avenues for investigating redox signaling [48,49]. As mentioned earlier, fluorescent dyes are non-specific, non-linear, and disruptive; therefore real-time quantification of ROS in living organisms is limited with redox-sensitive fluorescent probes. Use of *in vivo* redox sensors via either a hydrogen peroxide sensor protein coupled with a glutathione redox potential sensor (Hyper and Grx1-roGFP2, respectively) [48] or quantitative redox proteomics (OxICAT) [49] can overcome limitations of the fluorescent dye to provide quantification of ROS in an intact organism. HyPer and Grx1-roGFP2 are ratiometric biosensors that can be used to determine the oxidized-to-reduced ratios of H_2_O_2_ and GSSG/2GSH. Yellow fluorescent protein is inserted into the H_2_O_2_-sensitive regulatory domain of the bacterial transcription factor (OxyR-RD) [50], and oxidation of HyPer by H_2_O_2_ generates a disulfide bridge between OxyR-RD to induce changes in the fluorescence of the protein, which then can be quantified. Grx1-roGFP2 detects the glutathione redox potential when HyPer gets reduced by glutaredoxin-1 (Grx1) and GSH [51]. OxICAT is a redox proteomic technique, which monitors the *in vivo* oxidation status of several different redox-sensitive protein thiols. A thiol-reactive isotope-coded affinity tag (ICAT) differentially labels *in vivo* reduced and *in vivo* oxidized protein thiols. High performance liquid chromatography (HPLC) is then used to separate the ICAT-labeled peptides and mass spectrometry (MS) is used to identify the thiol-containing peptides for quantification of their *in vivo* oxidation status. Proteins are then characterized by oxidation status and subcellular location to provide information about temporal and spatial changes in cellular redox homeostasis. The use of genetically encoded redox-sensitive sensors and redox proteomics provides efficient methods to studying ROS in intact organisms.

### 3.3. Using Pharmacological Agents to Assess the Role of ROS in Dopamine Neuron Degeneration

Given the caveats in measuring *in vivo* ROS generation, researchers have instead examined sensitivity to oxidative stress, whereby increased sensitivity towards oxidative stress suggest an increased steady state level of ROS or a decreased ability to respond to ROS, which results in oxidative damage. Several compounds have been used to assess the sensitivity to oxidative stress with the most widely studied being the redox-cycling compounds, juglone [52–55], and paraquat [56–59]. Redox-cycling inducing molecules lead to generation of intracellular superoxide, and subsequent oxidative stress [60]. The application of pharmacological agents to attenuate ROS generation represents not only an indirect measurement of oxidative damage, but also exposes the worms to conditions that would normally not be encountered. It is possible that a worm may have increased ROS levels and show sensitivity towards the redox-cycling inducing molecule of choice, yet under normal conditions contains antioxidant defenses sufficient to detoxify all of the endogenous ROS produced. While the pharmacological agents for inducing oxidative stress are thought to act through ROS, in some instances, worms have been found to be sensitive to one form of oxidative stress, but not sensitive (or resistant) to another ROS generator [61–63]; therefore, experimenting with different pharmacological agents to produce the desired result may be necessary.

Typically, pro-oxidant molecules are administered to worms either during development or in young adult worms. When administered during development, sensitivity to oxidative stress is assessed by examining either the percentage of worms that are able to develop to adulthood or the furthest developmental stage obtained. Interpretation of results becomes a bit more complicated when the development time differs between the strains compared [64]. In order to obtain uniformity in comparison between strains, the slower developing strain will be exposed to the oxidative stress for a longer period. In addition to the aforementioned consideration, assays performed during development and adulthood may yield different results. Varying results could be due to high ROS production during development, which results in an increased sensitivity to oxidative stress, thus inducing the upregulation of antioxidant defenses and decreasing sensitivity to oxidative stress during data collection in adulthood. Therefore, examining sensitivity to oxidative stress at multiple developmental points is informative to these studies.

### 3.4. Methods to Quantify Oxidative Damage

Measuring levels of ROS production is not the only index of oxidative insult; quantifying the amount of oxidative damage via multiple methods can also provide valuable information in neurodegeneration studies. To measure oxidative damage, researchers have focused on either collecting products of lipid oxidation or quantifying damage to proteins. F_2_-Isoprostanes ((IsoPs), products of free radical-induced peroxidation of arachidonic acid)) are currently thought to be the most reliable marker of oxidative damage in humans [65,66]. The predominant polyunsaturated fatty acid (PUFA) in *C. elegans*, eicosapentaenoic acid (EPA), was also identified as an oxidative damage marker [67]; as such, measuring PUFA levels are presented as a viable approach for quantifying endogenous oxidative damage in *C. elegans* through a mass spectrophemetric-based assay of F_3_-IsoPs [68]. Other approaches to measure oxidative damage focuses on measuring protein carbonylation by derivatization with dinitrophenylhydrazine and detection with antibodies [69–71], or through detection of reactive lipid aldehydes, such as 4-hydroxynonenal (4-HNE) or isoketals (IsoK) with antibodies [72–74]. Lastly, transgenic green fluorescent protein (GFP) reporter *C. elegans* strains can be utilized as indirect measures of oxidative stress. Sensitivity towards juglone was assessed using a GFP transgenic reporter for the *C. elegans* cap “n” collar transcription factor SKN-1 (the mammalian NRF-2 homolog) target gene *gst-4* (glutathione-s transferase) and molecular determinants of SKN-1 activation has been studied through use of the *gst-4* reporter [55,75–77].

## 4. Oxidative Stress and PD-Associated Neurodegeneration in *C. elegans*

### 4.1. DJ-1

The *DJ-1/PARK7* gene encodes a protein of 189 amino acids, which forms a single 20 kDa domain homologous to the prokaryotic ThiJ family, a protein involved in biosynthesis of thiamine [78]. Different pathogenic mutations (exonic deletions, truncations, and homozygous and heterozygous point mutations) in *DJ-1* have been associated with rare forms of autosomal recessive, early-onset Parkinsonism [78,79]. Therefore, the biochemical function is of central importance in shedding light on disease pathogenesis. The most well-characterized *DJ-1* mutation, L166P, leads to protein destabilization and misfolding [78,80,81], and loss of dimerization necessary for functionality [82]. Several studies point out the role of DJ-1 in oxidative stress protection [83,84], mitochondrial function [85] and DAergic neuroprotection [86,87]. Additionally, it has been identified as a peroxiredoxin-like peroxidase [88] and has probable chaperone activity to prevent α-synuclein aggregation [82]. Recent studies identified DJ-1 as a transcription regulator modulating dopamine homeostasis-related genes [87]. The DJ-1 protein responds to oxidative stress with an acidic shift by oxidation of Cys106, and the oxidized form is shuttled from the cytoplasm to mitochondria [89]. The acidic isoform has been found in higher abundance in sporadic PD brains compared to normal brains [87,90]. Evidence suggests that the acidic isoform might act as a ROS scavenger through auto-oxidation [91]. Additionally, the active cysteine site is proposed to be involved in enzymatic activities (protease, glyoxylase) of DJ-1 and binding to biological macromolecules like RNA [92]. Recently, it has been shown that dopamine-derived quinones are responsible for impairing DJ-1 function by covalently modifying Cys106 [93]. While overexpression of DJ-1 protects against dopamine toxicity and oxidative stress, DJ-1 deficiency leads to increased ROS accumulation, oxidative insults and DA neurodegeneration [87,91,94,95]. Additionally, *DJ-1* mutations cause mitochondrial fragmentation [85]. DJ-1-deficient dopaminergic neurons display mitochondrial deficits, such as decreased complex I and II activity, accumulation of damaged mitochondria, and moderate oxidative stress [96]. DJ-1 is also suggested to have a key role in regulating the antioxidant capacity by regulating the expression of superoxide dismutase-1 and superoxide dismutase-3, as well as potentially facilitating the activation of the Nrf2 pathway [97]. Mice deficient in DJ-1 are hypersensitive to oxidative stress and DAergic neurodegeneration upon MPTP treatment [98].

Recent results have also shown an interaction between *DJ-1* and two other PD-associated genes *Parkin* and *PINK1*. Up-regulation of DJ-1 can rescue PINK1, but not Parkin, resulting in protection against oxidative stress [99]. DJ-1 is also believed to be involved in promoting Parkin translocation. Loss of DJ-1 results in an increased stress-induced Parkin recruitment and increased mitophagy due to the loss of its ability to control ROS generation in these mutants [100]. Overexpression of either PINK1 or Parkin rescues the fragmented mitochondrial phenotype seen in DJ-1 deficient cells, suggesting that DJ-1 is acting in parallel to the PINK1/parkin pathway. However, the possibility of a DJ-1-PINK1-parkin multi-protein complex remains controversial [101,102].

DJ-1 protein is ubiquitously expressed in most mammalian tissues, including the brain, and predominantly localizes in the cytosol but can also translocate to mitochondria [103,104]. While mammals contain a single DJ-1 homolog, some species, such as *Drosophila melanogaster* (DJ-1β and 1α) or *C. elegans* (DJR-1.1 and 1.2) contain two homologs with different tissue expression patterns. DJR-1.1 in *C. elegans* was detected in both the nucleus and cytoplasm of the intestinal cells, whereas DJR-1.2 is only expressed in the cytosol of head neurons [105]. The mutation of the conserved Cys106 site in DJR-1.1 in *C. elegans* results in abolished enzymatic activity [105]. Knocking down *djr-1.1* resulted in increased vulnerability to rotenone-induced toxicity, which was rescued by treatment with a combination of d-βhydroxybutyrate and tauroursodeoxycholic acid [106]. An additional function of DJ-1 in *C. elegans* is characterized by its glyoxalase activity. Detoxifying reactive glyoxals (α-oxoaldehydes) is of crucial importance, because they react with proteins to form advanced glycation end products, which have been implicated in the etiology of PD. Both of the DJ-1 homologs in *C. elegans* were characterized as glyoxylases, with DJR-1.1 being more efficient in protecting worms against glyoxals than DJR-1.2. *C. elegans* DJR-1.2 have also been shown to exert neuroprotective effects [105]. Furthermore, it has been demonstrated that the expression of DJR-1.2 increased dramatically when worms entered dauer stage. This expression was mediated by DAF-16, which is the *C. elegans* homolog of the mammalian FoxO protein that regulates entry into the dauer stage. Additionally, *djr-1.2* expression is likely to be regulated as part of the insulin signaling pathway, as confirmed by showing that *daf-2* (*e1370*) mutants, and not the *daf-2; daf-16* double mutants, show increased DJR-1.2 activity against glyoxals [107].

### 4.2. *Parkin* and *PINK1*

The *PARKIN* gene encodes for an E3 ubiquitin ligase, a component of the ubiquitin-proteasome system (UPS) to target substrate proteins for proteasomal degradation [108]. The protein is composed of 465 amino acids, and contains a ubiquitin-like domain that is responsible for substrate recognition, as well as RING finger domains that interact with other components of the UPS [109]. Brain *PARKIN* expression is distributed within basal ganglia structures, including the SN and caudate-putamen, but also with some expression in the cerebellum [110]. In addition to itself, Parkin has many substrates, including the synaptic vesicle-associated protein CDCrel-1 [108], α-synuclein [111], the α-synuclein-interacting protein synphilin-1 [112], and the membrane receptor Pael-R [113]. Parkin has also recently been shown to form an E3 ligase complex with DJ-1 and PINK1, two other proteins associated with PD [101]. Homozygous mutations found in *PARKIN* are responsible for nearly 50% of early-onset, familial forms of PD that abnormally present without Lewy body deposition [109]. *PARKIN* mutants show altered intracellular localization of Parkin, along with impaired substrate binding and enzymatic activity. Consequently, a functional effect of *PARKIN* mutations is an inability to degrade substrate proteins [114]. *PARKIN* knockout mice exhibit increased extracellular striatal dopamine (DA) concentration [115], while wildtype Parkin seems to increase cell surface expression of the dopamine transporter (DAT) and increase DA reuptake [116]. *PARKIN* knockout mice also have impaired synaptic plasticity [117], as Parkin seems to negatively regulate the strength and number of excitatory synapses [118]. Moreover, animal models expressing mutant *PARKIN* exhibit selective DAergic degeneration as well as hypokinetic deficits [119,120], as seen in PD cases. Interestingly, Mn exposure in cells was found to increase Parkin protein levels specifically in DAergic cells. Parkin also conferred protection from Mn-induced DAergic cell death *in vitro*, and was selectively redistributed to the perinuclear region in DAergic cells upon Mn exposure [121]. However, the mechanism behind these effects is still unclear and needs further investigation.

Another PD-associated gene that has been strongly tied to oxidative stress is *PINK1*, which spans eight exons and encodes for a 581-amino acid containing kinase known as PTEN-induced kinase 1 (PINK1). While not as common in mutational frequency as *PARKIN*, homozygous mutations in this mitochondrial-targeted kinase also result in a familial, early-onset form of PD [122]. Reduced striatal DAT binding has been seen in PD patients who possess a G309D substitution mutation in the *PINK1* gene [123], a mutation that occurs in a highly conserved region of the serine/threonine kinase domain [122]. Moreover, *PINK1* knockout mice show decreased dopamine release and impaired corticostriatal synaptic plasticity [124]. In the normal human brain, PINK1 is ubiquitously expressed in all cell types, with subcellular fractionation and immunohistochemistry studies confirming a punctate, localized expression to mitochondrial membranes [125]. Its kinase domain indicates a neuroprotective role via phosphorylation of specific mitochondrial proteins to regulate proper mitochondrial function [126]. Wildtype (WT) PINK1 can protect against MPP^+^-induced DAergic cell death in primary neurons [127]. Moreover, studies using DAergic cell line SH-SY5Y have found that wildtype PINK1 promotes the reduction of cytochrome c release from mitochondria, as well as lowering levels of several apoptotic caspases [128]. In fibroblasts extracted from patients carrying the G309D mutation, a battery of oxidative stress markers were measured to find elevated lipid peroxidation, as well as increased levels of MnSOD, oxidized (GSSG) glutathione, total glutathione, glutathione reductase (GR), and glutathione-*S*-transferase (GST). The *PINK1* mutant fibroblasts also showed decreased mitochondrial complex I activity that corresponded with a trend toward elevated superoxide production [129].

Interestingly, a key phosphorylation target of PINK1 is Parkin [130,131]. Initial, landmark findings show that Parkin and PINK1 work together in a common pathway to maintain mitochondrial integrity within DAergic neurons, and that Parkin functions downstream of PINK1 in this pathway [132]. Further studies found that the PINK1/parkin pathway in *Drosophila* actually promotes fission and inhibits fusion of mitochondria [133], and that Parkin translocation to damaged mitochondria with lowered mitochondrial membrane potential is dependent on PINK1 expression (and autophosphorylation [134]) that promotes aggregation of these mitochondria into the perinuclear region for autophagic elimination (“mitophagy”) [135–137]. More recent evidence has revealed the involvement of several novel modulators of this interaction, including the mitochondrial outer membrane protein, Mitofusin 2 (Mtfn2), as a PINK-1-mediated phosphorylation target that acts as a ubiquitination substrate for Parkin [138], and is required for proper axonal projections of midbrain DAergic neurons [139]. Additionally, the role of voltage-dependent anion channels (VDACs) has also come into light, with evidence showing their recruitment of Parkin to damaged mitochondria for proper autophagy [140]. While these studies represent a small proportion of the expansive PINK1-Parkin literature, such remarkable results signify that PD pathophysiology could arise from ineffective clearance and trafficking of defective mitochondria due to mutations in *PARKIN* or *PINK1*, ultimately resulting in neurodegeneration. However, the selectivity of this story in the context of DAergic neurons has yet to be resolved.

**Figure 2:**
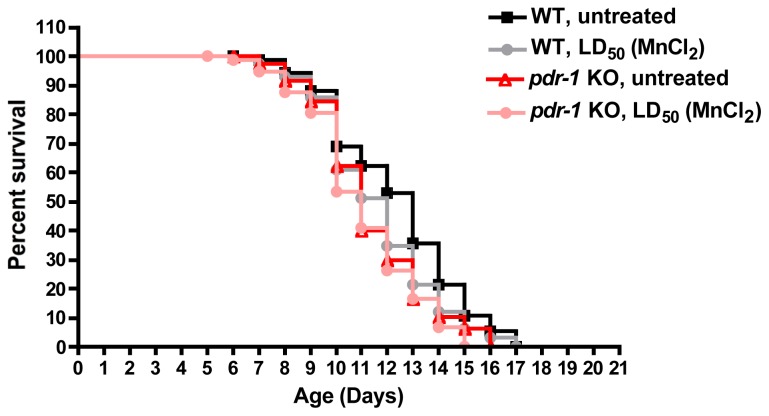
Decreased lifespan in *pdr-1* KO worms is exacerbated by Mn exposure. Two thousand five hundred L1 worms were treated with MnCl_2_ for 30 min, washed and then plated onto NGM plates spread with OP50 bacteria. Twenty worms were plated in triplicates per group. Worms were assessed for survival each day, and transferred to fresh plates every other day, until all worms had died. Wildtype worms: N2 strain; *pdr-1* KO: *pdr-1* (*gk448*) deletion. Compared to untreated WT animals, the other groups show a statistically significant leftward shift in lifespan (logrank test, *p* < 0.001), with one-way ANOVA analysis on median survival finding a significantly decreased lifespan in treated WT worms (*p* < 0.001) that is enhanced in the *pdr-1* KO animals (*p* < 0.001).

In *C. elegans*, *pdr-1* (PD related-1) is a homolog for *PARKIN* that shows conservation of its function as an E3 ubiquitin ligase. Similar to *PARKIN*, the *pdr-1* homolog is ubiquitously expressed in the worm, and shows high expression in both cell bodies and dendrites of neurons [141]. Similarly, the *PINK1* homolog in worms, *pink-1*, also shows conservation in having both cytoplasmic and mitochondrial localization, as well as its serine-threonine kinase domain. Moreover, in response to paraquat-induced ROS generation, *pink-1* deletion mutants exhibit shortened mitochondrial cristae and neuronal axon pathfinding defects [142]. The use of nematodes to study the role of these PD-associated genes in oxidative stress-mediated neurodegeneration is relatively novel. However, new evidence has shown that the oxidative stress and subsequent DAergic neurodegeneration in *C. elegans* may be selective towards particular toxicants. For example, compared to wildtype worms, *pdr-1* knockout worms show increased lethality and shortened lifespan upon exposure to methylmercury (MeHg) that correspond to increased ROS induction. However, the *pdr-1* mutants do not show the same dopamine-dependent behavioral deficits that wildtype worms do upon MeHg exposure [143]. On the other hand, these same *pdr-1* knockout worms also express increased lethality and shortened lifespan (Figure 2) upon Mn exposure, yet they do show enhanced Mn-induced DAergic neurodegeneration compared to wildtype worms [144].

It is important to note the role of *LRRK2*, or leucine-rich repeat kinase 2, a gene that is the most frequent cause of autosomal-dominant, late-onset PD [145,146]. Interestingly, this gene has also been shown to interact with the aforementioned autosomal-recessive genes. LRRK2 is predominantly found in the cytoplasm but can also associate with the mitochondrial outer membrane [147]. Pathogenic mutations result in increased kinase activity [147] that result in mitochondrial dysfunction [148] and neuronal degeneration [149]. Recent evidence has also found enhanced autophagic elimination of dendritic mitochondria and calcium dysregulation in mouse cortical neurons expressing mutant *LRRK2* [150]. This is interesting, as previous evidence has shown an interaction between Parkin and LRRK2 *in vitro* [149] and in a *Drosophila* model of PD [151], with Parkin able to protect against DAergic neurodegeneration induced by mutant *LRRK2* [152]. In *C. elegans*, the homolog for LRRK2 is known as LRK-1 and is required for the polarized localization of synaptic vesicle proteins between the axon and dendrites [153]. Loss of *lrk-1* increases vulnerability to mitochondrial inhibition as seen by a decrease in survival compared to WT worms [154], with human mutant *LRRK2* expression in worms resulting in increased protein oxidation and lipid peroxidation [155]. While interactions between *lrk-1* and other PD-associated genes in *C. elegans* have not yet been elucidated, it has been shown that *lrk-1* and *pink-1* have antagonistic roles in terms of toxin-induced stress responses and axonal outgrowth. The involvement of these proteins implicates altered kinase activity in PD pathophysiology, as kinase inhibitors were recently found to prevent and reverse mutated LRRK2-induced dopaminergic-specific behavioral deficits and DAergic neurodegeneration in *C. elegans* [156].

### 4.3. Nrf2

Nuclear factor erythroid 2-related factor 2 (Nrf2) is a basic leucine zipper transcription factor. Nrf2 protects mammals from oxidative stress and age-related diseases by regulating Phase II detoxification enzymes and some antioxidant genes [157–159]. Nrf2-deficient mice show lower basal levels of Phase II enzyme expression and lack the ability to induce them [145,160]. Nrf2 is ubiquitously expressed in a wide range of tissues and cell types and has been identified in many species including *Caenorhabditis briggsae*, *Drosophila melanogaster*, *Xenopus laevis*, *Mus musculus*, and humans. In *C. elegans*, SKN-1 has been identified as a homolog of Nrf2 [161]. SKN-1 is expressed in both the intestine and in ASI neurons, and the differentially localized SKN-1 isoforms provide distinct biological functions. Expression of SKN-1 in ASI neurons is required for lifespan extension in response to dietary restriction [162]. In contrast, oxidative stress in the intestine is required to direct SKN-1 to the nuclei and promote expression of protective genes [163]. In general, activation of Nrf2 can be induced by direct modifications of Keap1 (Kelch-like ECH-associated protein 1), or phosphorylation that promotes Nrf2 stabilization [164,165]. Oxidative stress can disrupt this interaction, resulting in nuclear translocation of Nrf2 and the induction of cytoprotective genes [166,167]. However, a putative KEAP1 homolog has not been described in *C. elegans*.

In general, SKN-1 activity in *C. elegans* results in the upregulation of numerous genes involved in detoxification to increased stress resistance, and in the downregulation of genes that decrease lifespan [161,162]. Global gene-expression profiles with and without *skn-1* (via RNAi) identified several target genes of SKN-1 under both basal conditions and oxidative stress that are directly involved in stress-related processes, including: GST (glutathione *S*-transferase), UGT (UDP-glucuronosyl/glucosyl transferase), and other Phase II genes [168–170]. For example, SKN-1 mediates stress-induced transcription of *gst-4* (glutathione transferase-4), which has been monitored in *C. elegans* using strains expressing a transcriptional GFP reporter (p_gst-4_::GFP). In fact, upon exposure to H_2_O_2_, sodium azide and the redox cycler juglone, these worms been shown to induce *gst-4* [55,75,171]. Moreover, an adaptation to oxidative stress tolerance dependent on SKN-1 has been shown in *C. elegans*. H_2_O_2_-induced adaptation to oxidative stress was strongly dependent on an SKN-1-mediated increase in both proteolytic activity and expression of the 20S proteasome. A novel role of SKN-1 has been identified in regulating synaptic transmission at neuromuscular junctions in response to stress. Additionally, SKN-1 has been determined as regulator in innate immunity suggesting its involvement in immunosenescence and providing a crosstalk between pathogenic stress signaling and the oxidative stress response [172,173].

As *skn-1* mutants show reduced resistance to oxidative stress and shortened lifespan, SKN-1 overexpressing worms show a higher resistance towards oxidative stress and increased lifespan [161,163,174]. Consequently, in response to environmental toxins, while worms overexpressing SKN-1 showed a resistance to Mn toxicity, worms containing mutated *skn-1* exhibit a hypersensitive phenotype. An observed nuclear relocation of SKN-1 in ASI neurons, consistent with a Mn-induced increase in oxidative stress, further illustrated that SKN-1, at least in part, mediates Mn toxicity in worms [30]. Evidence also suggests that age affects the cytoprotective transcriptional pathway, as indicated by a decrease in the hormetic response to juglone with increased age of worms [55].

## 5. Conclusions and Future Directions

While the literature on PD-associated genes and disease pathophysiology continues to grow, the enigmatic mechanisms behind the characteristic selectivity of cell loss, mitochondrial dysfunction and increased oxidative stress remain unclear. The utilization of a model system, such as *C. elegans*, allows for ease in high-throughput screening and unbiased forward genetics approaches, in addition to faster genetic studies investigating the role and/or interplay of specific genes associated with the disease. While their transparent bodies allow for *in vivo* visualization of DAergic neurodegeneration in the nematode, further studies must be conducted to understand the selectivity associated with DAergic cell loss in PD. The list of PD-associated genes includes major redox modulators, implicating a significant role of oxidative stress in promoting the cell death seen in the disease. However, the interplay between several PD genes represents a complex scenario, which is further complicated by the role of environmental toxicants in triggering and facilitating the disease’s progression.

For example, the more recent mitophagy theory connected PINK1 and Parkin proteins as co-players, working together to promote a healthy environment, free of oxidant-induced damaged mitochondria that would otherwise wreak havoc on the oxidative stress status of the cell. However, when these genes are mutated, this system is impaired, resulting in increased oxidative stress that results in DAergic neurodegeneration in the SN. What causes the selective injury within these cells remains unknown. One hypothesis posits that there are brain regions that have inherently higher frequencies of mitophagy. Thus, when proteins such as Parkin and PINK1 are impaired, these brain regions (potentially the SN) become more vulnerable to oxidative damage. This is likely, as the basal ganglia are highly metabolically active [175], and consequently, may need more efficient mitochondrial turnover and trafficking to promote an environment free of oxidative stress.

However, the question remains: why does this affect the DAergic neuronal subtype specifically in the SN, and not other cells? Dopamine itself is considered to be a strong oxidant that also produces auto-oxidization products [176]. In combination with brain region-specificity of proper mitophagy, there could also be a role that dopamine itself plays in interacting with other toxicants to produce damaging quinones and other highly reactive radicals that cause cell death within those neurons. This could coincide with the specific localization of transporters (such as DAT or the divalent metal transporter DMT1 [177]) that have been shown to transport many of the neurotoxins associated with PD. Additionally, impairment of Nrf2 in itself would be damaging to these cells, as this represents one of the primary cellular defense pathways against oxidative stress. However, in combination with exposure to a toxicant and within the environment of highly oxidative dopamine, neurodegeneration would be inevitable without a fully functioning antioxidant defense system.

Therefore, PD pathophysiology remains intricate, with a complex system of proteins and defense mechanisms that may be working together to protect against oxidative stress in neurodegeneration. Current treatment options seem to focus on the effects of dopamine supplementation and cell replacement therapy. However, these therapeutic routes are riddled with their own flaws, including detrimental side effects and the lack of sustainability in drug delivery [178]. It will be crucial that future investigations of PD have a circuit level focus on the pathophysiology found in the SN to understand how PD-associated proteins alter normal function. For example, while the use of cell lines and cultured neurons allows a glimpse into DA-specific effects, co-culture systems are more representative of neuronal networks within the brain that may be involved together in PD pathophysiology. Moreover, the newer world of optogenetics may provide an interesting avenue, even within the *C. elegans* model system, to further examine the role of PD-associated proteins within the context of intact, neural networks.
